# Abnormal Longitudinal Strain in Takotsubo Cardiomyopathy: A Case Report

**DOI:** 10.7759/cureus.24289

**Published:** 2022-04-19

**Authors:** Paramjit Kaur, Syed S Fatmi, Darius Aliabadi, Saikiran Mandyam, Sebastian T Tosto

**Affiliations:** 1 Internal Medicine, Southeast Health Medical Center, Dothan, USA; 2 Cardiology, Southeast Medical Center, Dothan, USA

**Keywords:** acute coronary syndrome, speckle tracking, echocardiogram, global longitudinal strain, takotsubo cardiomyopathy

## Abstract

In recent years, the prognostic utility of global longitudinal strain (GLS) has evolved significantly in the evaluation and management of a wide array of cardiac conditions. Various studies have described the use of GLS in the evaluation of various cardiac pathologies, including heart failure, aortic stenosis, and acute myocardial infarction. Evaluation utilizing speckle-tracking echocardiography (Echo) has been shown to be sensitive in the assessment of global and regional myocardial function. In that context, GLS can be used as a surrogate marker of myocardial function, especially in cases of acute myocardial infarction. Although GLS has been shown as a sensitive marker for myocardial ischemia, it has been a significantly underutilized modality in the evaluation of Takotsubo cardiomyopathy (TC); an acute myocardial stress reaction, which can mimic acute coronary syndrome on presentation. With this case report, we present a case of left ventricular TC with abnormal longitudinal strain affecting the entirety of the apex and all three major coronary artery distribution territories. Our case report illustrates how GLS can be a sensitive marker for myocardial dysfunction in cases of TC. The extent of abnormality and distribution of strain has a pathognomonic ‘evil eye’ appearance, which was described in previous studies and is consistent with TC. GLS may help identify patients with TC prior to proceeding with left heart catheterization and would be significantly beneficial in TC and may have further implications on the overall prognosis and management of TC in the future.

## Introduction

Takotsubo cardiomyopathy (TC) or broken-heart syndrome [1] is an acute stress reaction and is considered mostly a reversible condition. TC is primarily defined as systolic dysfunction of the left ventricle, absence of obstructive coronary artery disease or acute plaque rupture, abnormal electrocardiogram (EKG), and/or troponin in the absence of other etiology of myocarditis or conditions such as pheochromocytoma [2]. 2D echocardiography (Echo) is the standard and primary utilized imaging modality in establishing the diagnosis of TC [3]. Recently, with the evaluation of longitudinal strain, it has been employed, and its clinical utility has been documented in multiple studies [4,5]. However, it is still an underutilized imaging modality to diagnose and predict prognosis in patients with TC. With this case report, we present a case of TC which highlights the significant incremental changes in strain pattern and how it has great clinical and diagnostic value in the diagnosis of TC.

## Case presentation

A 58-year-old female with a history of hypertension, hyperlipidemia, hypothyroidism, meningioma, and shingles initially presented to the ED with complaints of acute chest pain. The patient was noted to be non-compliant with her medication regimen. She had started going through significant stresses in her life secondary to concerns about the health condition of a family member. The patient was hypertensive on presentation without any other abnormalities on vital signs. Initial EKG showed normal sinus rhythm without any acute ST changes. Initial high sensitivity troponin was noted to be 2113.4 ng/L (normal reference range <15.0 ng/L), which was trended and peaked at 3111.9 ng/L (normal reference range <15.0 ng/L). She was admitted for acute non-ST elevation myocardial infarction and was started on therapeutic anticoagulation with enoxaparin, 1 milligram/kg twice a day. The patient stated that her chest pain started after getting stressed while visiting a family member who was suffering from a serious medical condition.

**Figure 1:**
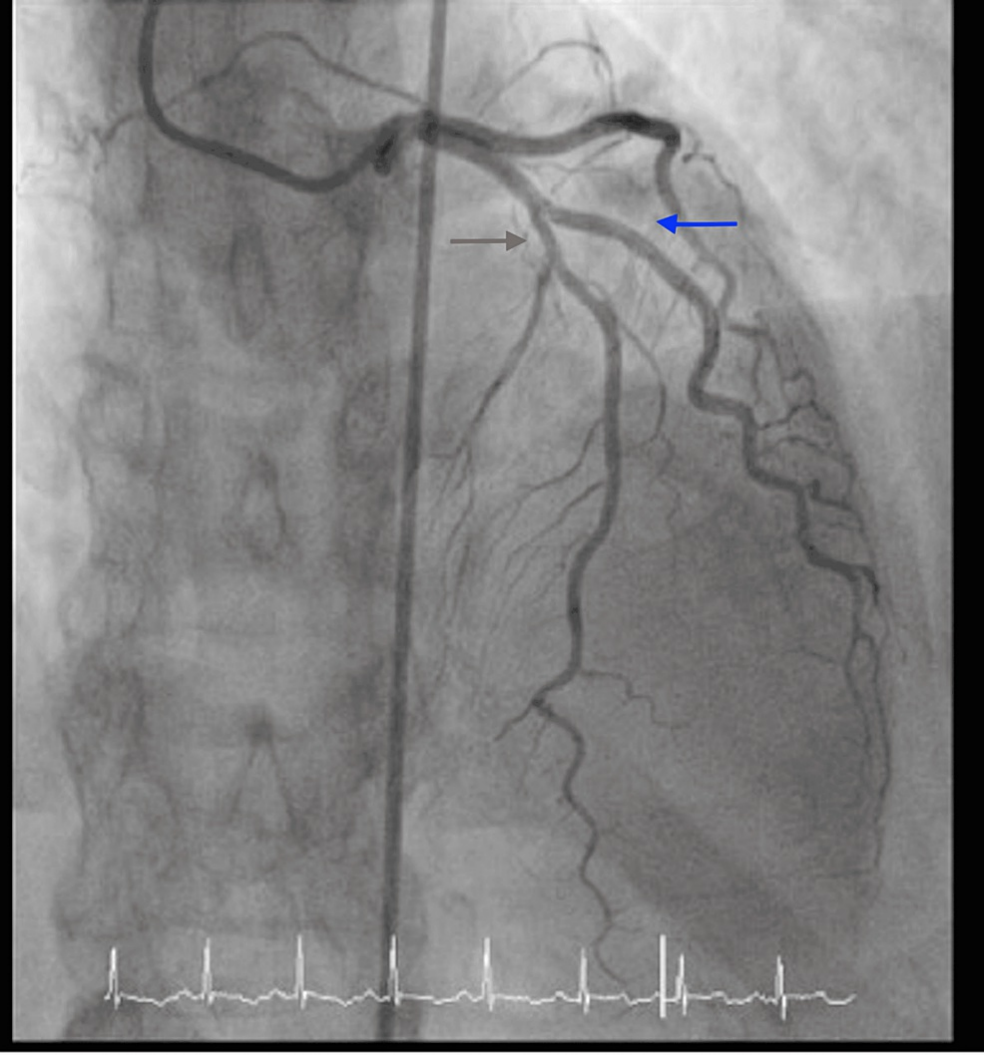
Cardiac catheterization sequence depicting left coronary circulation Left anterior descending marked with the blue arrow and left circumflex coronary artery marked with the red arrow, without any identified obstructive lesions.

**Figure 2:**
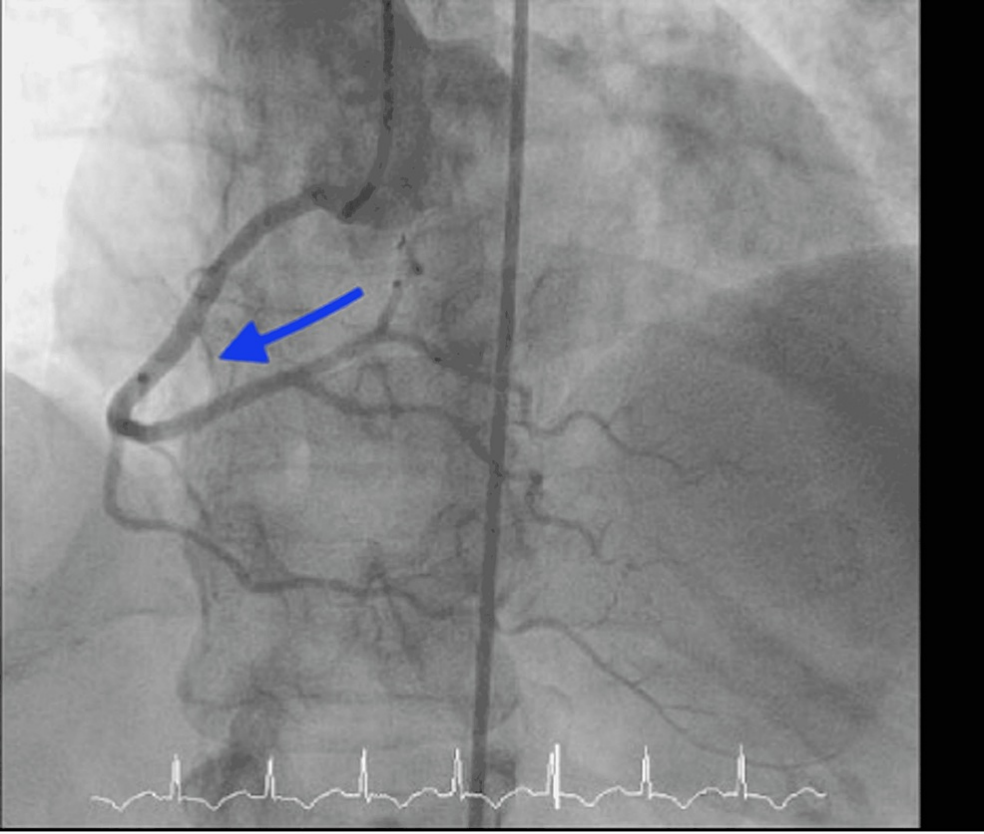
Cardiac catheterization sequence depicting right coronary artery The right coronary artery without obstructive lesion is marked with the blue arrow.

**Figure 3:**
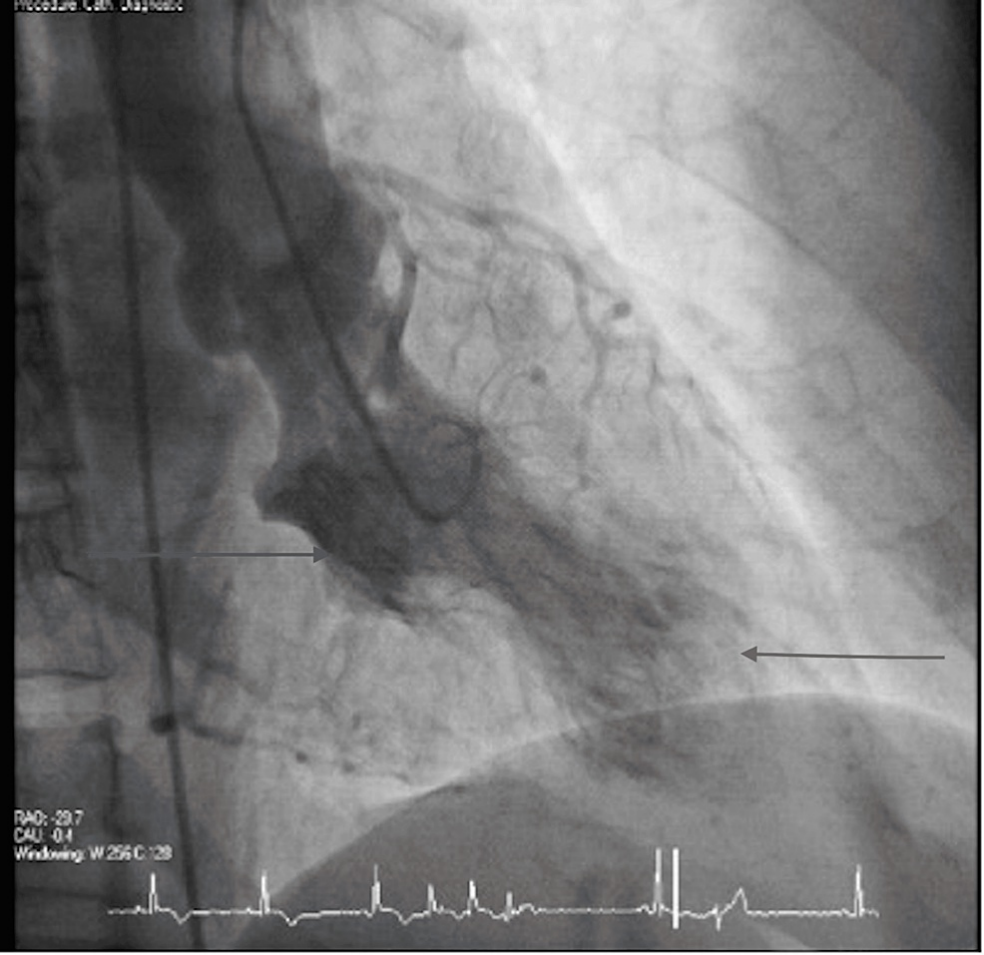
Cardiac catheterization sequence depicting octopus appearance Cardiac left heart catherization image with contrast during systole showing base of the heart, top arrow showing good contraction and akinesis of apex marked with bottom arrow, depicting octopus’ appearance.

**Figure 4:**
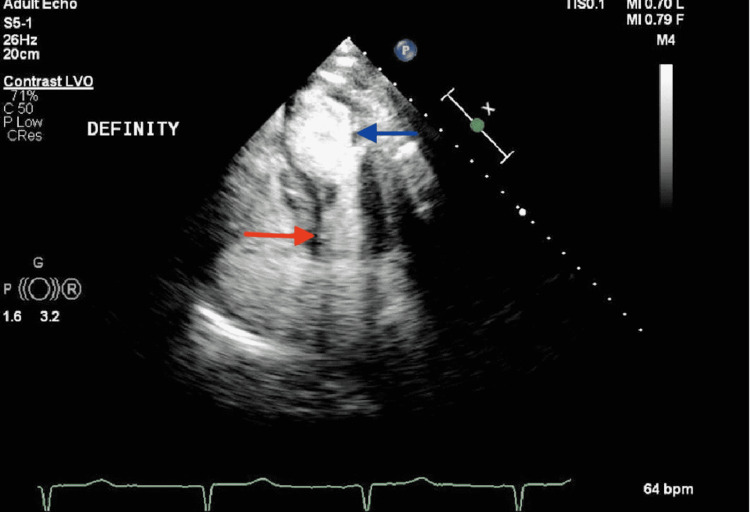
2D Echo on a patient with Takotsubo cardiomyopathy. 2D echocardiography (Echo) with contrast, imaged during systole depicting normal contraction at the base depicted by the red arrow and severe hypokinesis at the apex depicted by the blue arrow.

**Figure 5:**
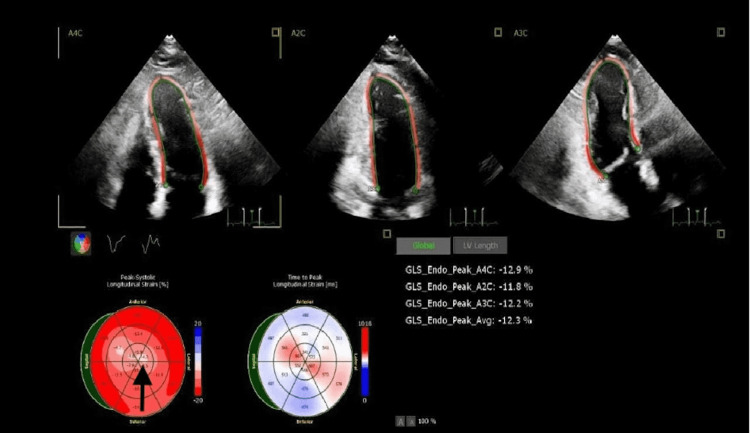
Global longitudinal strain with LV base and apex, depicting "evil eye" appearance Global longitudinal strain with "evil eye" appearance depicted by the black arrow. On strain sequence, peak systolic longitudinal strain was less than -18%, suggestive of perfusion defect in three coronary artery territories (LAD, LCX, RCA) LV - left ventricular, LAD - left anterior descending artery, LCX - left circumflex artery, RCA - right coronary artery

After presenting to the hospital, the patient was taken for cardiac catheterization the following day, which showed no significant stenosis in the left anterior descending and left circumflex, right coronary arteries, as shown in Figures 1 and 2 below, but apical ballooning was noted with apical akinesis during left heart catheterization as shown in Figure 3. Subsequently, a traditional 2D Echo with contrast was obtained, which revealed mild left ventricular (LV) hypertrophy, and extensive apical wall motion abnormality was noted. Figure 4 demonstrates contractility of the base of the heart with systole, apical ballooning of the apex is seen. No thrombus was seen in LV apex with DEFINITY contrast (Lantheus Medical Imaging, Billerica, Massachusetts), and left ventricular ejection fraction (LVEF) was noted to be 35%. Limited Echo with longitudinal strain was noted to have an abnormal longitudinal strain at -12%. Regional strain analysis, as noted in Figure 5, demonstrated severely abnormal strain involving the apex, as well as apical septal, apical lateral, anterior apical, and inferior apical regions. No abnormality on strain was noted on the base of the left ventricle.

## Discussion

Echocardiography is the primary modality used for the evaluation of most structural abnormalities of the left ventricle, along with the diagnosis of TC and various other cardiac conditions. Utilization of Echo with strain has been limited in the past for evaluation of TC, and strain Echo has been an underutilized modality. After analysis of existing data, it is quite evident that most patients with TC will clinically recover within one year, but it is still not clear when the strain will recover [3]. Significant data and follow-up echo imaging with strain is needed to assess the strain values after clinical recovery and improvement of symptoms.

Non-ST elevation myocardial infarction (NSTEMI) and TC may have a lot of clinical similarities on presentation and can have similar findings on initial workup, including elevation of troponins, particularly in cases of NSTEMI and Takotsubo cardiomyopathy [6]. A 2D Echo with global longitudinal strain (GLS) is a noninvasive modality that can be employed early in the course to differentiate between these two conditions based on temporal strain changes along with anatomic strain patterns. Improvement in strain pattern without any intervention can also be utilized to differentiate the two conditions. Studies have shown patients with TC had early improvement in left ventricular GLS when compared with patients with obstructive coronary lesions [7].

With this unique case report, we have documented abnormal strain involving the apex as well as apical septal, apical lateral, anterior apical, and inferior apical regions in a patient presenting to our facility with Takotsubo cardiomyopathy. In our presentation, we also documented normal free wall strain, which was progressively worsened to the apical region with normal strain at the base in a circular motion, not seen in a specific coronary artery distribution [8]. One important finding on the strain pattern on Echo is the anatomical distribution, which is supplied by multiple coronary arteries and is not specific to the vascular distribution of one coronary artery, which is more commonly seen in the ischemic presentation. Evaluation with strain pattern echo in patients presenting with NSTEMI or elevated troponin is significantly beneficial for stratification and guiding further management [2,3,9].

## Conclusions

Patients presenting with suspected Takotsubo cardiomyopathy and troponin elevation should undergo 2D Echo with the evaluation of GLS for early diagnosis and management, which would lead to improved outcomes and faster recovery. TC presentation can mimic other cardiac conditions, including NSTEMI, and requires prompt evaluation treatment, and implementation of an early 2D Echo with strain images can help avoid urgent cardiac catheterization for these patients. Longitudinal strain and speckle tracking is a widely available noninvasive modality, which is easy to obtain without any logistical difficulties. This is critical in patients presenting with Takotsubo cardiomyopathy, a condition that mimics acute coronary syndrome. Although elective catheterization in these patients can be performed, utilization of strain images on Echo can stratify the acuity of cardiac catheterization.
